# Multiple neuroprotective features of *Scutellaria pinnatifida*–derived small molecule

**DOI:** 10.1016/j.heliyon.2020.e04737

**Published:** 2020-08-28

**Authors:** Soha Parsafar, Zahra Nayeri, Farhang Aliakbari, Farshad Shahi, Mehdi Mohammadi, Dina Morshedi

**Affiliations:** Department of Bioprocess Engineering, Institute of Industrial and Environmental Biotechnology, National Institute of Genetic Engineering and Biotechnology, Tehran, Iran

**Keywords:** Neuroinflammation, Neurotoxins, Neobaicalein, PD's omics data, Systematic bioinformatics analysis, Cytotoxicity, Biochemistry, Health sciences, Information science, Network analysis

## Abstract

Parkinson's disease (PD) is one of the most prevalent neurodegenerative disorders with no precise etiology. Multiple lines of evidence support that environmental factors, either neurotoxins or neuroinflammation, can induce Parkinsonism. In this study, we purified an active compound, neobaicalein (Skullcapflavone II), from the roots of *Scutellaria pinnatifida* (*S. pinnatifida*). Neobaicalein not only had protective impacts on rotenone-induced neurotoxicity but in glial cultures, it dampened the inflammatory response when stimulated with lipopolysaccharide (LPS). Neobaicalein had high antioxidant activity without any obvious toxicity. In addition, it could raise the cell viability, decrease early apoptosis, reduce the generation of reactive oxygen species (ROS), and keep the neurite's length normal in the treated SH-SY5Y cells. Pathway enrichment analysis (PEA) and target prediction provided insights into the PD related genes, protein-protein interaction (PPI) network, and the key proteins enriched in the signaling pathways.

Furthermore, docking simulation (DS) on the proteins of the PD-PPI network revealed that neobaicalein might interact with the key proteins involved in PD pathology, including MAPK14, MAPK8, and CASP3. It also blocks the destructive processes, such as cell death, inflammation, and oxidative stress pathways. Our results demonstrate that neobaicalein alleviates pathological effects of factors related to PD, and may provide new insight into PD therapy.

## 1 Introduction

Parkinson's disease (PD) is the second-most prevalent neurodegenerative disorder and the most common form of Parkinsonism with severe chronic symptoms. The interaction between environmental and genetic factors has an essential role in the pathogenesis of PD. The exposure to environmental neurotoxins such as pesticides and excess metals is a significant contributor to the development of PD and other forms of Parkinsonism [1, 2, 3]. One of the most well-known toxins is rotenone, a natural compound used as a pesticide [4], which can pass through the blood-brain barrier (BBB) [5]. It causes apoptotic cell death of tyrosine hydroxylase-positive neurons in substantia nigra (SN) of rats [6]. It induces aggregation of αSN, Aβ, and Tau in rotenone-treated mice, inhibits mitochondrial complex I, and eventually leads to dopaminergic neurodegeneration [7, 8, 9]. Rotenone binds to tubulin at the colchicine-site [10], which disturbs its structure, and depolymerizes microtubules [11]. It also induces morphological apoptotic features in dopaminergic cells [12, 13]. The use of rotenone in the rat's model of PD has shown the generation of proteinaceous inclusions in some dopaminergic neurons that cannot be found in the 6-OHDA and MPTP models [14]. Overall, it could be an acceptable neurotoxic agent for developing a good model of PD.

Moreover, internal factors are also known as key participants in the onset, development, and progression of PD. The main internal factors are neuroinflammation, oxidative stress, mitochondrial dysfunction, α-synuclein aggregation, and calcium hemostasis dysregulation [15]. Microglial activation, which usually accompanies the release of pro-inflammatory cytokines and also ROS production, associates with dopaminergic neuronal death in PD [16]. Postmortem studies show elevated pro-inflammatory cytokines such as TNF-α, IL-1β, IL-2, IL-4, IL-6, IL-10, IFNγ in the striatum, and activated glial cells within the SN of PD patients [17]. Microglial activation and increased levels of cytokines have been observed in the mice and adult rats treated with LPS, and the investigation has shown that direct injection of LPS into the SN of mice causes TH^+^ cell loss [18, 19]. Given the importance of neuroinflammation in PD, developing the compounds with anti-inflammatory effects can have therapeutic benefits. In this context, small phytochemical molecules, especially flavonoids, could be potent modulators [20] as they have antioxidant, anti-inflammatory, and anti-amyloidogenic properties [21, 22, 23]. A recent study showed that small molecules could protect mitochondria in neurodegenerative diseases [24]. These metabolites seem to have a multi-target mechanism of action and interact with different signaling cascades, which can lead to neuronal survival [25, 26].

Furthermore, they can cross the BBB [27], induce neurogenesis, and exert free radical scavenging properties [28]. One source of flavonoids is the *Scutellaria* genus, which belongs to the *Lamiaceae* family [29]. Extracts and isolated active constituents from *Scutellaria* have antioxidant, anti-inflammatory, and neuroprotective properties [30]. *Scutellaria pinnatifida* (*S. pinnatifida*), locally known as *Boshghabi* [31], is one of the Iranian species of the *Scutellaria* genus. This genus is renowned for its medical benefits for insomnia, cancer, hepatitis, allergy, and arteriosclerosis [32, 33]. We have previously shown that its flavonoid contents, particularly those extracted with dichloromethane (DCMex), have the highest inhibitory activity against the aggregation of α-synuclein, and show antioxidant properties [34].

Taking all the noteworthy biological features of *S. pinnatifida* into account, we investigated the chemical composition of DCMex, isolated neobaicalein (skullcapflavone II), and for the first time evaluated its neuroprotective effects on rotenone-induced cell toxicity and LPS induced inflammation. Due to the multiple biological functions of the small molecules, the in silico experiments, virtual screening, and reverse docking methods can simulate their action and shed light on their interaction with new targets [35, 36]. Here, different methods such as pathway enrichment analysis, reverse docking, and MD simulation were employed to anticipate the mechanism of multiple neuroprotective activities of neobaicalein and offer a convenient perspective towards therapeutic drugs for PD.

## 2 Experimental procedures

### 2.1 Reagents and chemicals

2′,7′-dichlorodihydrofluorescein diacetate (DCFH-DA), 1,1-diphenyl-2-picrylhydrazyl (DPPH^•^), 3-(4,5-dimethylthiazol-2-yl)-2,5-diphenyl tetrazolium bromide (MTT), Griess reagent and rotenone were purchased from Sigma-Aldrich. SH-SY5Y neuroblastoma cell line was acquired from Pasteur Institute (Iran). All salts and organic solvents were purchased from Merck (Darmstadt, Germany). The cell culture medium (DMEM-F12, High glucose), fetal bovine serum (FBS), and antibiotics were from GibcoBRL (Life Technologies, Paisley, Scotland). Standard blood agar plates were from Mehrazmalab (Iran), Silica gel (60–100 mesh, pore size 60 Å), and TLC plates from Sigma-Aldrich (Germany). Neonatal *Wistar* rats were purchased from Pasteur Institute, Iran.

### 2.2 Plant materials

*S. pinnatifida* was collected in August 2018 from the road of Asalem to Khalkhal area (Gilan, Iran). The plant has been deposited at the Herbarium of TARI (Iran) with a voucher specimen (No. 107147). It was dried at room temperature, and the roots were ground by an electric blender to increase its surface extent. The air-dried powder roots were kept in a cold and dry place until they were used for the extraction.

### *2.3* *S. pinnatifida* extraction and fractionation procedures

We decided to choose the dichloromethane extract (DCMex) for more comprehensive analysis as it has the most protective effect on the α-synuclein fibrillation and neurotoxicity, among other *S. pinnatifida* fractions [34]. The air-dried powder roots (170 g) were soaked in 1 L *n*-hexane solvent at room temperature to eliminate the hydrophobic compounds. After 48 h, the whole n-hexane extract was removed, and then the extraction was progressed on the root residues by dichloromethane (DCM) solvent at room temperature. After 72 h, the whole extract was filtered, and the solvent was evaporated by a rotary evaporator under vacuum at 25 °C. The resulted extract (DCMex) was kept at -20 °C for future studies.

#### 2.3.1 Purification of neobaicalein

##### 2.3.1.1 Evaluation of the extract by thin-layer chromatography (TLC)

The content of the DCMex was analyzed with TLC. Each sample was spotted on a TLC plate with *n*-hexane/ethyl acetate (1.5:1) as a mobile phase.

##### 2.3.1.2 Purification of neobaicalein using column chromatography

We used the cylindrical glass column (length = 20 cm, diameter = 1.5 cm) to purify the compounds of DCMex. The concentrated DCMex was run through a silica gel column chromatography with a gradient of n-hexane–ethyl acetate as eluent. The column washing initially started with low polarity solvents (n-hexane: ethyl acetate ratio of 9 to 1) to a medium polarity and finally ended by adding 100% ethyl acetate. Twenty fractions were collected, and the purity of the components in each fraction was evaluated by TLC. Therefore, the same spots on the TLC plate were mixed and subjected to structure elucidation by ^1^H-NMR. The final compound was dissolved in DMSO and kept at -20 °C.

### 2.4 Free radical scavenging activity of neobaicalein

100 μL of neobaicalein with different concentrations (0.2, 0.3, 0.5, and 0.8 mM) was mixed with 200 μL of DPPH^•^ (final concentration = 100 μM dissolved in methanol), and the mixture was incubated in the dark for 60 min [37]. The scavenging activity of neobaicalein to reduce DPPH^•^ was measured at 517 nm with a plate reader (microplate spectrophotometer, Epoch 2, BioTek company, Gen5 software, USA). The experiment was carried out in triplicate, and methanol was used as blank. The percentage of antioxidant activity was calculated as follow:

(1) $$\text{\%}\;\text{of}\;\text{antioxidant}\;\text{activity }=\frac{\text{OD}\left(517\;\text{nm}\right)\;\text{of}\;\text{control}-\text{OD}\left(517\;\text{nm}\right)\text{of}\;\text{sample}}{\text{OD}\left(517\;\text{nm}\right)\text{of}\;\text{control}}\times 100$$

where “control” is the untreated DPPH^•^, and “sample” is DPPH^•^ in the presence of different concentrations of neobaicalein.

### 2.5 Hemolysis assay

To assess the biocompatibility of neobaicalein, a 100 μL of the compound)final concentration 0.5 mM( was spread on the surface of a blood agar plate and incubated for 24 h at 37 °C. As a control, an equivalent of 0.5 McFarland of *Staphylococcus aureus* was cultured on the same culture medium and incubated for 24 h at 37 °C. The lack of detectable hemolysis was regarded as a mark of biocompatibility [37, 38].

### 2.6 The effects of rotenone on SH-SY5Y cell line in the presence and absence of neobaicalein

MTT, ROS, flow cytometry, and the morphology of neurons were investigated to assess the neurotoxicity effect of rotenone against dopaminergic SH-SY5Y cells and the neuroprotective effect of neobaicalein against rotenone. The cells were cultured in DMEM-F12 supplemented with 10% FBS, 100 U/mL penicillin, and 100 μg/mL streptomycin at 37 °C in a humidified atmosphere with 90% humidity and 5% CO_2_. Rotenone and neobaicalein were dissolved in dimethyl sulfoxide (DMSO, 100%). Different concentrations of neobaicalein were added to the cell cultures one hour before adding rotenone (500 nM).

#### 2.6.1 Assessment of the cell metabolic activity

There were four distinct groups: control group (the cells with no treatment), neobaicalein group (the cells treated with different concentrations of neobaicalein), rotenone-group (the cells treated with 500 nM of rotenone), and rotenone/neobaicalein group (the cells pretreated with neobaicalein for one hour followed by exposure to 500 nM rotenone). The MTT colorimetric assay was used to evaluate the mitochondrial metabolism of viable cells. The oxidoreductase enzymes in living cells can convert the tetrazolium MTT (yellow dye) to insoluble purple formazan. SH-SY5Y cells were seeded at a density of 10^4^_cells/well_ in 96-well plates (200 μL) incubated for 24 h and treated with 20, 50, 150, 250 μM of neobaicalein to evaluate the possible cytotoxic effect. Afterward, the cells were incubated with rotenone (500 nM) in the presence or absence of neobaicalein for 24 h. The media were then replaced with fresh pre-warmed media supplemented with 10% (v/v) MTT stock solution (5 mg/mL in PBS). After 4 h of incubation, 100 μL DMSO was added to dissolve the formazan crystals. To assess the mitochondrial metabolic activity, the absorbance was read at 570 nm (microplate spectrophotometer, Epoch 2, BioTek company, Gen5 software, USA) and the percentage of the viable cells was calculated as follow [37]:

(2) $$\text{Cell}\;\text{viability}\left(\text{\%}\right)=\frac{\left(\text{Absorbance}\;570\;\text{nm}(\text{treated}\;\text{cells}\right)}{\left(\text{Absorbance}\;570\;\text{nm}(\text{untreated}\;\text{cells}\right)}\times 100$$

#### 2.6.2 Flow cytometry to detect early apoptosis and late apoptosis/necrosis

The cells were seeded in 6-well plates at a density of 5×10^5^_cells/well_, treated with neobaicalein (150 μM) in the absence or presence of 500 nM of rotenone and incubated for 24 h. The cells were then trypsinized (0.25% trypsin, one mM EDTA) and centrifuged for 3 min at 2000 rpm. The pellets were rinsed with PBS, resuspended in the binding buffer containing FITC-conjugated Annexin V and PI, incubated for 5 min in the dark, and then loaded on a Gallios Flow Cytometer (Beckman Coulter, CA, USA). The percentages of apoptotic or late apoptotic/necrotic cells were analyzed using Flowing Software v.2.5 [38].

#### 2.6.3 The measurement of intracellular ROS

To assess reactive oxygen species (ROS) activity within the cells, the cell-permeant fluorogenic reagent DCFH-DA was employed. After diffusion into cells, DCFH-DA is deacetylated by esterases, which are later oxidized by ROS into a highly fluorescent compound DCF [39]. SH-SY5Y cells were seeded in 96-well plates with densities of 2 × 10^4^_cells/well_ (200 μL) and incubated in the incubator (5% CO_2_, 90% humidity) for 24 h. Subsequently, the cells were treated with 50, 150, 250 μM of neobaicalein in the absence or presence of rotenone (500 nM), incubated for 6 h in a humidified atmosphere, and DCFH-DA (15 μM) was added to each well. After 45 min incubation in the dark, the cells were trypsinized, and their fluorescence emission intensity was measured at 527 nm (excitation: 495 nm).

#### 2.6.4 The differentiated SH-SY5Y cell neurite length analysis

SH-SY5Y cell line is able to undergo neuronal maturation. The cells can be differentiated into neuron-like cells with neurites outgrowth in response to all-trans-retinoic acid (RA) [40]. Therefore, differentiated SH-SY5Y cells have been used as appropriate *in vitro* cell model to study the mechanism of rotenone neurotoxicity. SH-SY5Y cells were grown in 12-well plates to a density of 4×10^4^_cells/well_ and then treated with RA dissolved in DMEM-F12 (2% FBS) to a final concentration of 10 μM, incubated for eight days (the medium was replaced every two days) [41]. Subsequently, neobaicalein (150 μM) in the absence or presence of rotenone (500 nM) was added to each well. Within 12 h of incubation, the length of the neurites was analyzed using the Image J software (NIH, USA). The images were taken at 20x magnification with 4608 × 2592 pixels. By changing the pixels to μM, the images were calibrated, and then Neuron J [42] plugin Image J software was used to measure the length of neurites by tracing them [43].

### 2.7 NO^•^ production in the primary mixed glial cells of the rat brain

The rat pups of *Wistar* strain, less than 72-hour-olds, were used for the preparation of mixed glial cell cultures. The whole process was done according to the guidelines of the National Institute of Genetic Engineering and Biotechnology Ethics Committee (ethics number: IR.NIGEB.EC.1398.10.18. A). Briefly, the brain was separated from the cranium, and meninges/vessels were removed carefully using forceps. Next, the tissue was mechanically triturating by pipetting, and the cell suspension was placed into a tissue culture flask. The cells were grown in DMEM high glucose medium supplemented by 20% FBS, 100 U/mL penicillin, and 100 μg/mL streptomycin at 37 °C and 5% CO_2_. The medium was changed the next day after the culture establishment and at five-day intervals. In 5–7 days, the astrocytes form a confluent cell layer, and microglia and some oligodendrocytes grow on top of the astrocytic layer. In order to induce inflammation on mixed glial cells, the cells were trypsinized and seeded at a density of 30×10^4^_cells/well_ in a 6-well plate. LPS (10 μg/μL) conducted stimulation of astrocytes and microglia on mixed glial cells. To evaluate the protective effect of neobaicalein, one hour prior to LPS exposure, 150 μM of the compound was used for co-treatment with LPS (10 μg/μL). After 48 h of treatment, NO^•^ production was assessed by indirect measurement of nitrite concentration using colorimetric Griess assay in a 96-well plate. This assay relies on a diazotization reaction that was initially described by Griess in 1879 [44]. Initially, 50 μL of the cell supernatant was mixed with the 25 μL of 1% sulphanilamide, and incubated for 5 min. Subsequently, 25 μL of 0.1% N-1-naphthyl ethylenediamine dihydrochloride (NED) was added, and the samples were incubated for 5 min in the dark. The absorbance was then measured by a microplate reader at 540 nm. Standards of sodium nitrite in the range of 20–200 μM were used to calibrate the assay validation.

### 2.8 Target prediction and toxicity profile of neobaicalein

#### 2.8.1 Network analysis for PD related genes

To predict the effect of neobaicalein on PD, we first designed a PD network to identify the key proteins and related molecular pathways. To this end, the DisGeNET database (http://www.disgenet.org/) was used to access the genes associated with PD. This platform collects information from four different sources, including Online Mendelian Inheritance in Man (OMIM), UniProt/SwissProt (UNIPROT), Pharmacogenomics Knowledge Base (PharmGKB) and Comparative Toxicogenomics Database (CTD).

In the next step, the protein-protein interaction (PPI) network of the related genes was plotted using the Search Tool in the Retrieval of Interacting Genes database (STRING, http://string.embl.de/). We used the STRING app in Cytoscape and applied the confidence score ≥0.7 and the maximum number of interactors = 0 as the cut off criterion. We calculated Radiality, BottleNeck, Betweenness, Stress, and Closeness parameters by cytohubba plugin to determine the most important proteins involved in the PD-related PPI network. The top 10% common genes acquired based on these parameters were considered as hub genes and, the related sub-network was extracted for these genes.

#### 2.8.2 Functional and pathway enrichment analyses

Gene ontology and pathway enrichment analyses were investigated by DAVID (Database for Annotation, Visualization, and Integrated Discovery, https://david.ncifcrf.gov/) based on the key proteins derived from the PD-related PPI network. Then, the related signaling pathways were extracted.

#### 2.8.3 Druglikeness and toxicity profile

Lipinski *et al.* formulated specific properties, known as the Rule of Five (RO5), to determine if the compound could be accepted as a drug [45]. To this end, MolSoft (https://molsoft.com/mprop/) was used to evaluate the neobaicalein properties based on Lipinski's rule. Also, PreADMET (https://preadmet.bmdrc.kr/) and ADVERPred (http://www.way2drug.com/adverpred/) [46] were applied to assess its toxicity and probable side effects, respectively.

#### 2.8.4 Molecular docking

A docking study was carried out by AutoDock Tools (ADT, 1.5.6) to investigate and anticipate the interaction of neobaicalein with the key proteins in the most significant pathways derived from enrichment analysis. The neobaicalein structure was retrieved from the PubChem database (https://pubchem.ncbi.nlm.nih.gov/). To acquire the optimized geometry of the neobaicalein structure, the webserver ATB, (Automated force field Topology Builder, http://compbio.biosci.uq.edu.au/atb), was used and 3D structures of the proteins were obtained from the RCSB PDB database (https://www.rcsb.org/). To apply the PDB data of proteins to ADT, nonessential chains, water, and ligand molecules, if present, were removed from the structure of proteins. In addition, Kollman charges and polar hydrogens were added to the structure files.

Furthermore, rotatable bonds of neobaicalein were indicated, gasteiger charges were added, and non-polar hydrogens were merged in the ADT environment. To create the map files for flexible atoms in the active sites, AutoGrid 4.0 was applied. We used ADT to determine the Docking Parameter File (DPF). To this end, the proteins were retained rigid during each docking study, and the docking run was performed by the Lamarckian Genetic algorithm, with 200 as the total number of runs. AutoDock 4.0 was used for docking simulation, and the 2D and the 3D binding interactions were studied with the Discovery Studio Visualization (DSV).

#### 2.8.5 Molecular dynamics

The Protein-Ligand complex dynamic simulations were carried out for proteins with the lowest binding energy to check the stability of docked conformation. All molecular dynamics (MD) simulations were performed for 50 ns using GROMACS package 5.1 by the GROMOS9643a1 force field. The topology parameters of the protein and ligands were obtained from Gromacs and Dundee PRODRG server, respectively. The Simple Point Charge (SPC) water model was used to develop the solvated systems, and corresponding ions for each simulation were added to neutralize the systems. Energy minimization of the docked complexes was achieved using the steepest descent algorithm at 1000 steps, and the system equilibration was performed under NVT and NPT ensembles at 300 k for 100 ps. In the end, the MD production run was carried out at 300 K temperature and 1 bar pressure.

### 2.9 Data analysis and statistics

The experiments were carried out as triplicates, and the data was stated as mean ± SD. SPSS software v.25.0 was used to study the statistically significant differences between the control and experimental groups. Unpaired Student's t-test and one-way ANOVA were used to analyze the significant differences between the groups and within the groups, respectively. P-value < 0.05 was considered as significant.

## 3 Results

### 3.1 Neobaicalein was purified from the roots of *Scutellaria pinnatifida*

**Figure 1:**
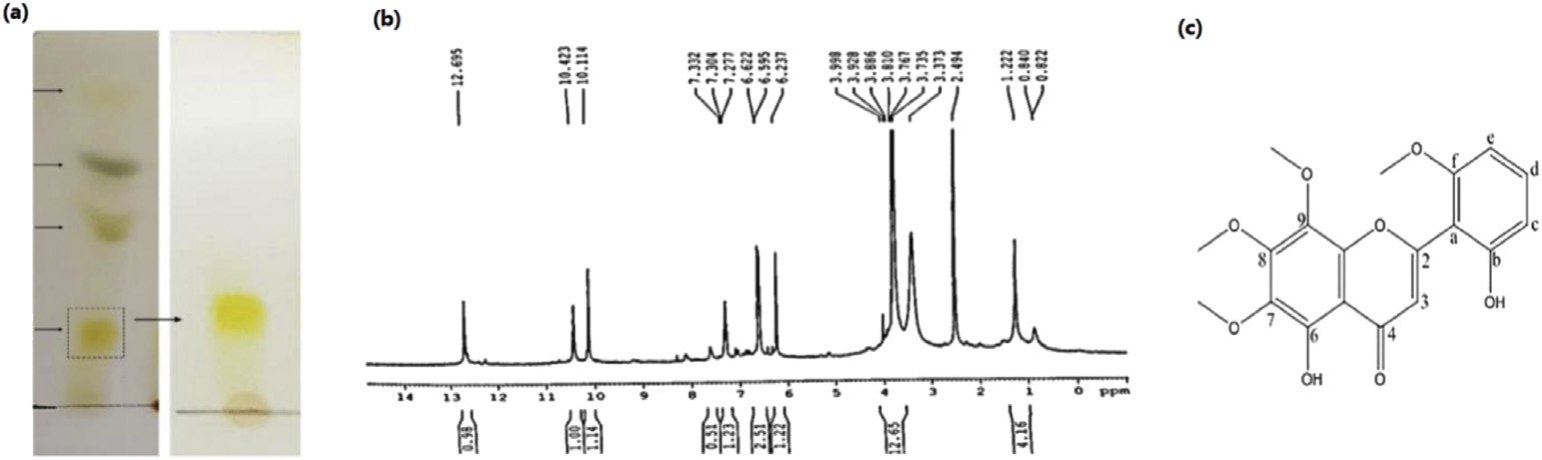
(a) Isolation pattern of DCMex (left) and neobaicalein (right) on TLC. (b) ^1^H-NMR spectrum of neobaicalein: ^1^H-NMR (DMSO, 300 MHz) δ: 12.7 (1H, s, OH-6), 10.4 (1H, s, OH-b), 7.3 (1H, dd, J = 8.4, 8.1 Hz, H-d), 6.6 (2H, m, H-c, H-e), 6.2 (1H, s, H-2), 3.8 (12H, s, O–CH3-7,8,9,f). (c) The chemical structure of neobaicalein. The full non-adjusted TLC image is provided in the supplementary material (Figure S1).

The initial analysis of DCMex through TLC showed four distinct bands (Figure 1a). Among different fractions that were achieved through purification by column chromatography, those with the same bands on TLC were mixed and further purified. This purification yielded 12 mg of a yellow-colored purified metabolite, related to the yellow-colored band on TLC (RF = 0.5, efficiency: 12%) (Figure 1a). The ^1^H-NMR analysis revealed the chemical structure of this metabolite (Figure 1b). The physical and spectral data of the isolated compound was identical to neobaicalein [47]. Neobaicalein (Skullcapflavone II) (Figure 1c) is a flavone with the chemical name of 5,2′-Dihydroxy-6,7,8,6′-tetramethoxyflavone and molecular weight of 374.345 g/mol. The detail of ^1^H-NMR analysis is mentioned in the caption of Figure 1.

### 3.2 Neobaicalein showed free radical scavenging activity with biocompatibility character

**Figure 2:**
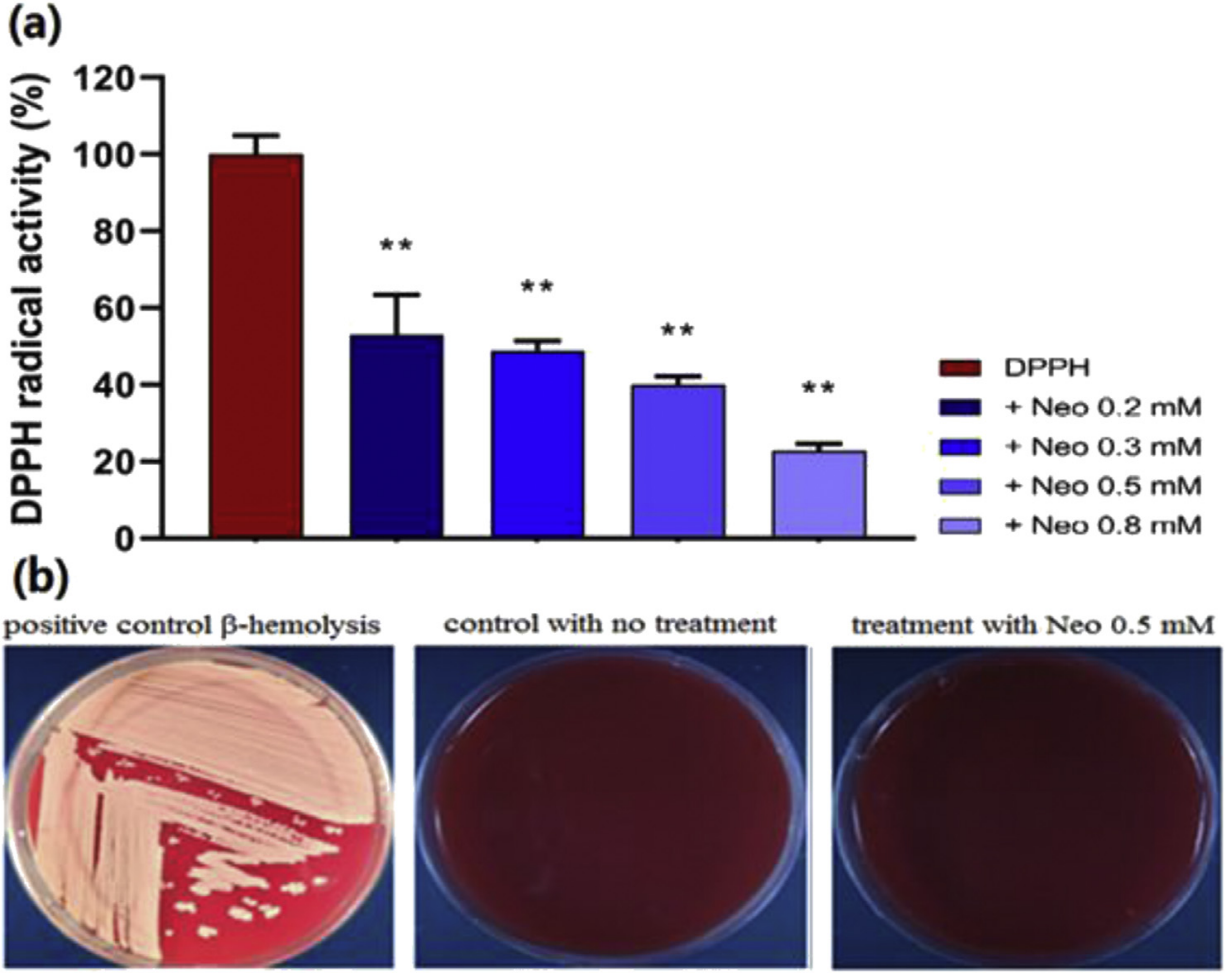
The antioxidant activity and biocompatibility of neobaicalein. (a) The scavenging activity was assessed using DPPH^•^. Neobaicalein showed dose-dependent free radical scavenging activity (∗∗ P-value ≤ 0.01 indicates statistically significant differences between the control and the treated samples). (b) Hemolysis assay was carried out to evaluate the biocompatibility. Neobaicalein was spread onto the surface of the blood agar medium. After 24 h of incubation, no hemolysis was detected in comparison with the β-hemolysis positive control (*Staphylococcus aureus*).

Treating DPPH^•^ radicals with different concentrations of purified neobaicalein, diminished the radicals significantly in a dose-dependent manner (Figure 2a). However, besides the radical scavenging properties of herbal compounds, some can be toxic to biological systems. Therefore, the biocompatibility of neobaicalein was assessed through the hemolysis assay. The results showed no hemolysis (γ-hemolysis) upon the spreading of neobaicalein (0.5 mM) on the blood agar media (Figure 2b).

### 3.3 Neobaicalein significantly protected the treated cells against rotenone neurotoxicity

#### 3.3.1 MTT assay

**Figure 3:**
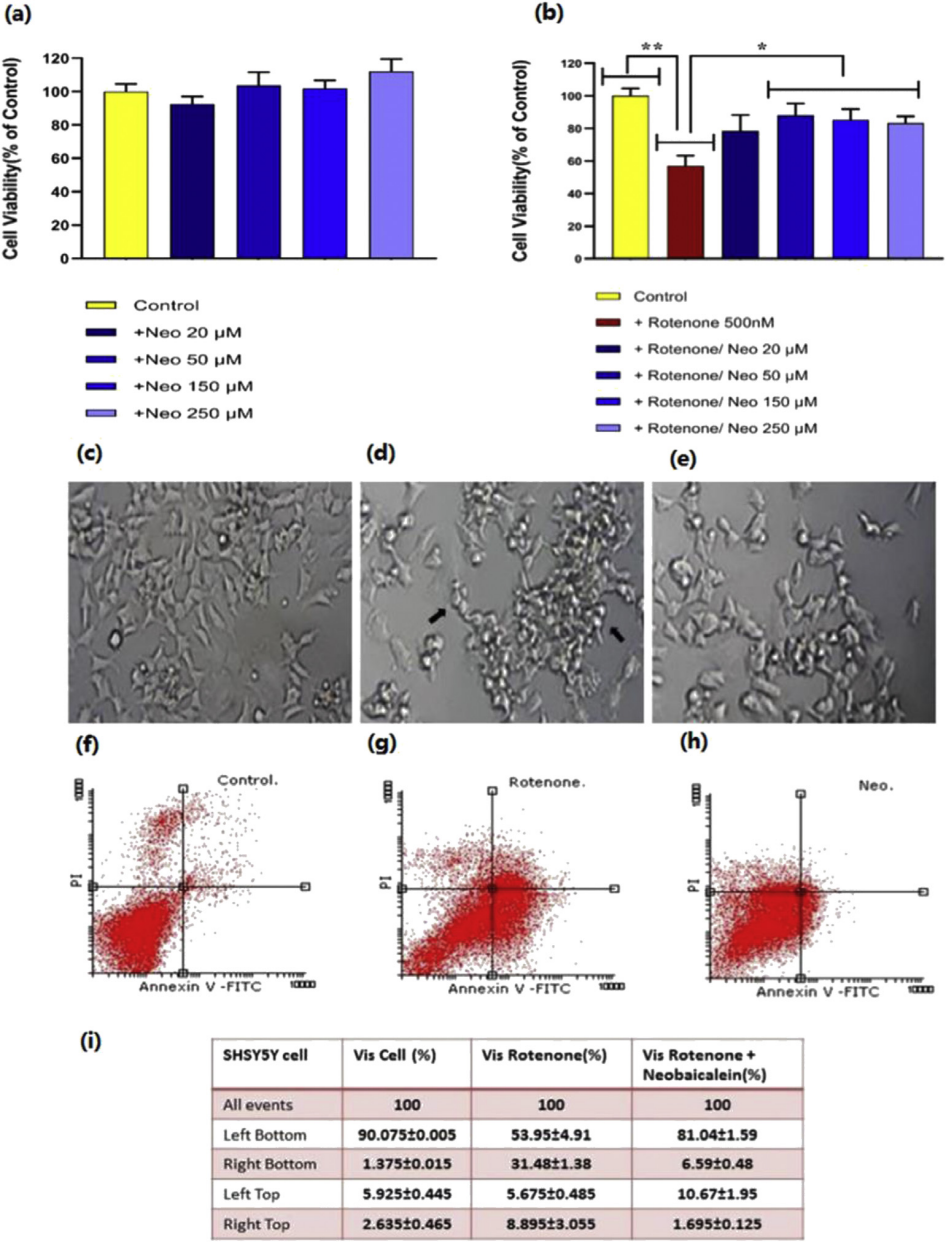
The effect of neobaicalein (Neo) on the viability of the rotenone treated SH-SY5Y cells evaluated by MTT and flow cytometry assays. (a) Different concentrations of neobaicalein (20, 50, 150, and 250 μM) did not induce cell death after 24 h of incubation. (b) Rotenone induced cell death and pre-treatment with neobaicalein, increased cell viability significantly (∗ P-value < 0.05, ∗∗ P-value ≤ 0.01). The morphology of (c) control cells, (d) rotenone treated cells, and (e) neobaicalein/rotenone treated cells. Black arrows show the morphological changes in (d) rotenone-treated cells. Flow cytometry shows the amount of the early apoptosis, late apoptosis/necrosis of (f) control cells, (g) rotenone treated cells, and (h) neobaicalein/rotenone treated cells. In diagrams, the lower left is for live cells, lower right for early apoptotic cells, upper left for necrotic cells, and upper right for late apoptotic/necrotic cells. (i) The table shows the average percentage of the cell count in each quadrant as mean ± SD.

The effect of neobaicalein on the neuronal cell survival was tested in the presence and absence of well-known neurotoxic agent rotenone. The viability of SH-SY5Y cells treated with different concentrations of neobaicalein (20, 50, 150, 250 μM) did not reduce significantly. However, rotenone (500 nM) induced significant cell death after 24 h of treatment (P-value < 0.01). On the other hand, pre-treatment with neobaicalein before adding rotenone increased cell viability by 34 % noticeably (P-value < 0.05) (Figure 3a, b).

#### 3.3.2 Flow cytometry assay

The main type of cell death induced by rotenone, as seen in neurodegeneration phenomena, was apoptosis. Also, in the presence of neobaicalein (150 μM)/rotenone (500 nM), the rate of early apoptosis, as well as late apoptosis/necrosis, decreased significantly (Figure 3f–h).

#### 3.3.3 ROS production assay

**Figure 4:**
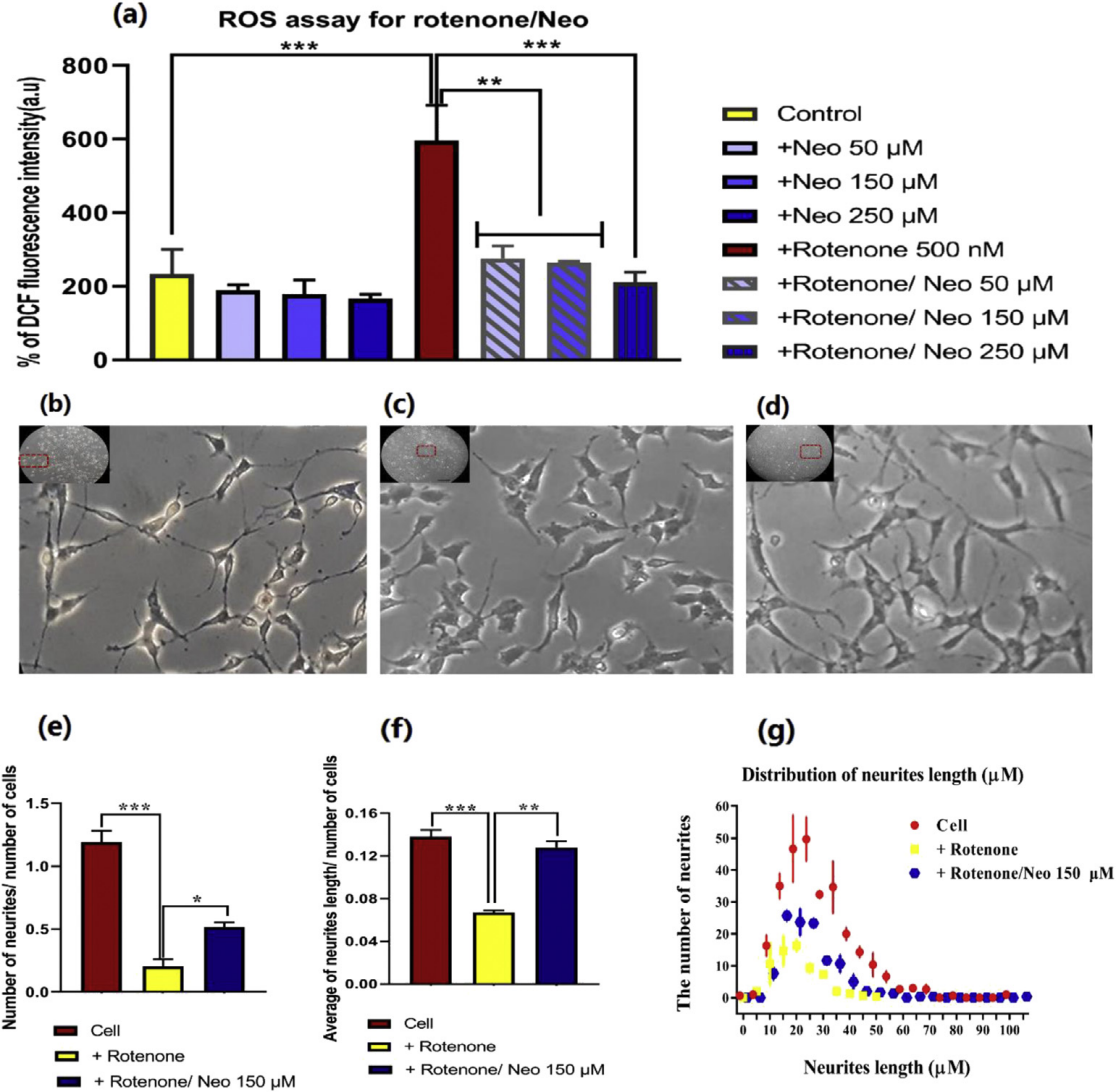
(a) The amount of ROS levels in SH-SY5Y cells treated with different concentrations of neobaicalein alone (50, 150, 250 μM) and in the presence of 500 nM rotenone detected by DCF fluorescence intensity. The morphology of differentiated SH-SY5Y cells and their neurites in (b) the cells with no treatment, (c) the cells treated with rotenone, and (d) the cells treated with rotenone/neobaicalein (inserts are the whole lens of the camera, and part of it is magnified). (e) The number of neurites, (f) the average of neurite length, and (g) the distribution of various neurite lengths were measured using image J. Scale bar, 100 μm. ∗P-value ≤ 0.05, ∗∗P-value ≤ 0.01, ∗∗∗P-value ≤ 0.001, (N = 3, Mean ± SD).

The result of the DCFH-DA assay demonstrates that ROS increased significantly in the rotenone-treated SH-SY5Y cells (P-value <0.001) and different concentrations of neobaicalein moderated its elevation, significantly (P-value <0.001) (Figure 4a).

#### 3.3.4 Neurite length measurement

Outspreading of neurites in neurons is crucial for neuronal activities such as neurotransmitters recruitment. Inhibition of axonal outgrowth and neurite elongation is attributed to numerous neurodegenerative pathologies [48]. In our study, following 8 days of RA usage, the neurites had an extensive outgrowth, demonstrating the differentiation property of SH-SY5Ycells (Figure 4b). Nevertheless, treatment with rotenone significantly influenced the length of neurites (Figure 4c). However, exposing the differentiated SH-SY5Y cells to neobaicalein before treatment with rotenone sustained the natural morphology of the neurons with extended neurites (Figure 4d). Different neurites parameters, including number (Figure 4e), the average of neurite lengths (Figure 4f), and the distribution of various neurite lengths (Figure 4g), were evaluated in SH-SY5Y cells in the presence and absence of rotenone or rotenone/neobaicalein. Neobaicalein restored the number of neurites, the average of neurite length, and their prevalence in rotenone treated differentiated SH-SY5Y cells (Figure 4e–g).

### 3.4 Neobaicalein alleviated the inflammation response of the primary mixed glial cells

**Figure 5:**
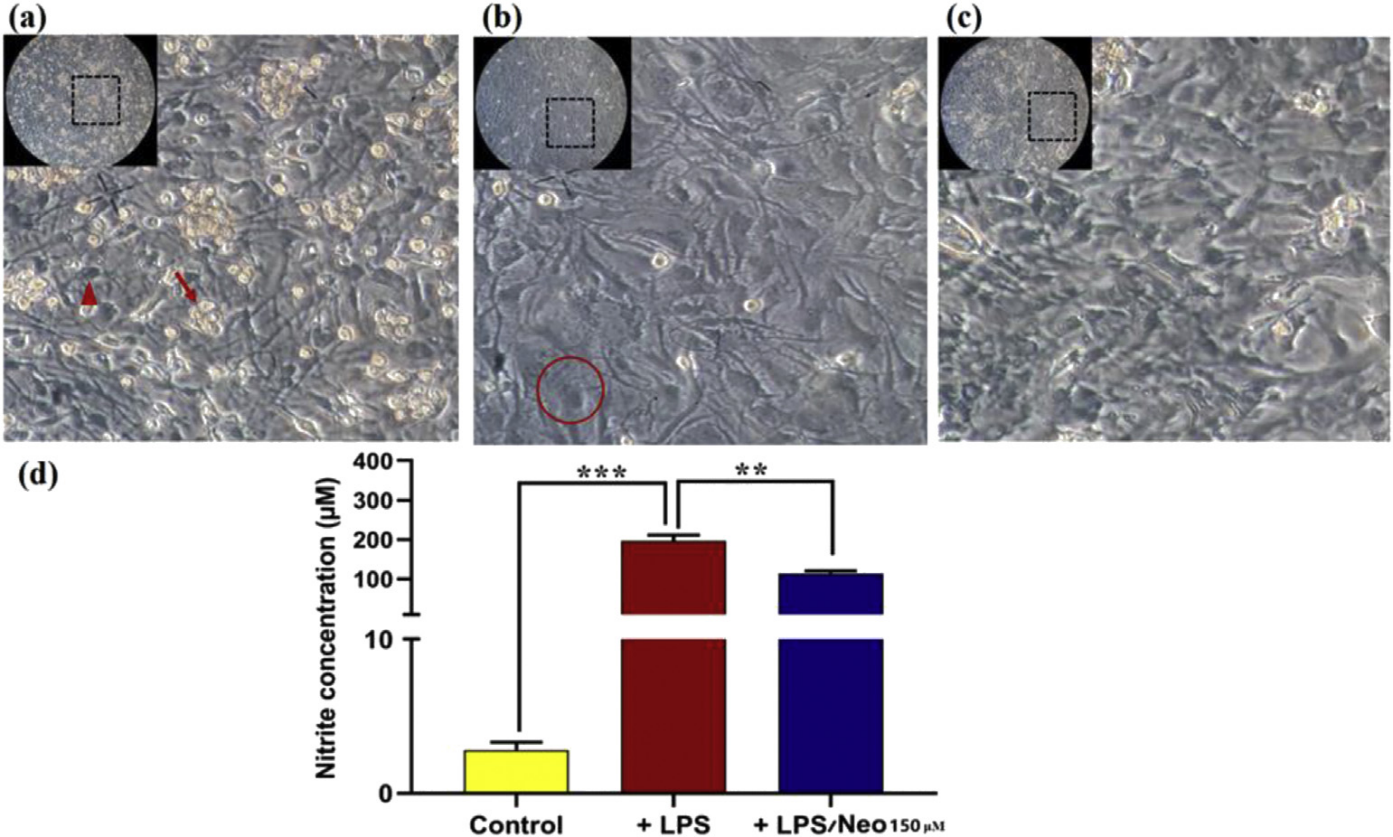
NO^•^ production in LPS-induced glial cells in the presence or absence of neobaicalein. (a) The morphology of mixed glial cultures (control) shows the basal layer of astrocytes. The dark-field oligodendrocyte precursor cells (triangle) adhere to the top of astrocytes, and small bright-field microglia (arrow) are rounded and floating. (b) The LPS-stimulated cells show different morphology. Most of them lose their spindle shapes, become flattened, and are not branched anymore (circle). (c) The natural morphology of the cells in the presence of neobaicalein/LPS is better preserved than the LPS-stimulated one (inserts are the whole lens of the camera, and part of it is magnified). (d) Nitrite level in the primary mixed glial cells after 48 h of treatment was measured using Griess reagent at 540 nm based on a standard curve of sodium nitrite. Values indicate the mean ± SD of three independent experiments. The symbols ∗∗ and ∗∗∗ indicates significance between the control group and experimental groups at P ≤ 0.01 and P ≤ 0.001, respectively.

We also assessed the effect of neobaicalein on the activity of glial cells, since inflammation plays a crucial role in the progression of neurodegenerative disorders. Based on the indirect measurement of NO^•^ by Griess assay, a significant increase in nitrite level was detected after 48 h of incubation with 10 μg/μL of LPS compared to the untreated cells (P-value ≤ 0.001). However, pre-incubation with 150 μM of neobaicalein, showed significantly less increase in nitrite concentration (P-value ≤ 0.01) (Figure 5).

### 3.5 Neobaicalein has potential inhibitory activities against death pathways in PD according to *in silico* studies

Systematic bioinformatics prediction was employed to gain insight into the molecular mechanisms involved in the neuroprotective activities of neobaicalein.

#### 3.5.1 PPI network construction and network analyses

**Figure 6:**
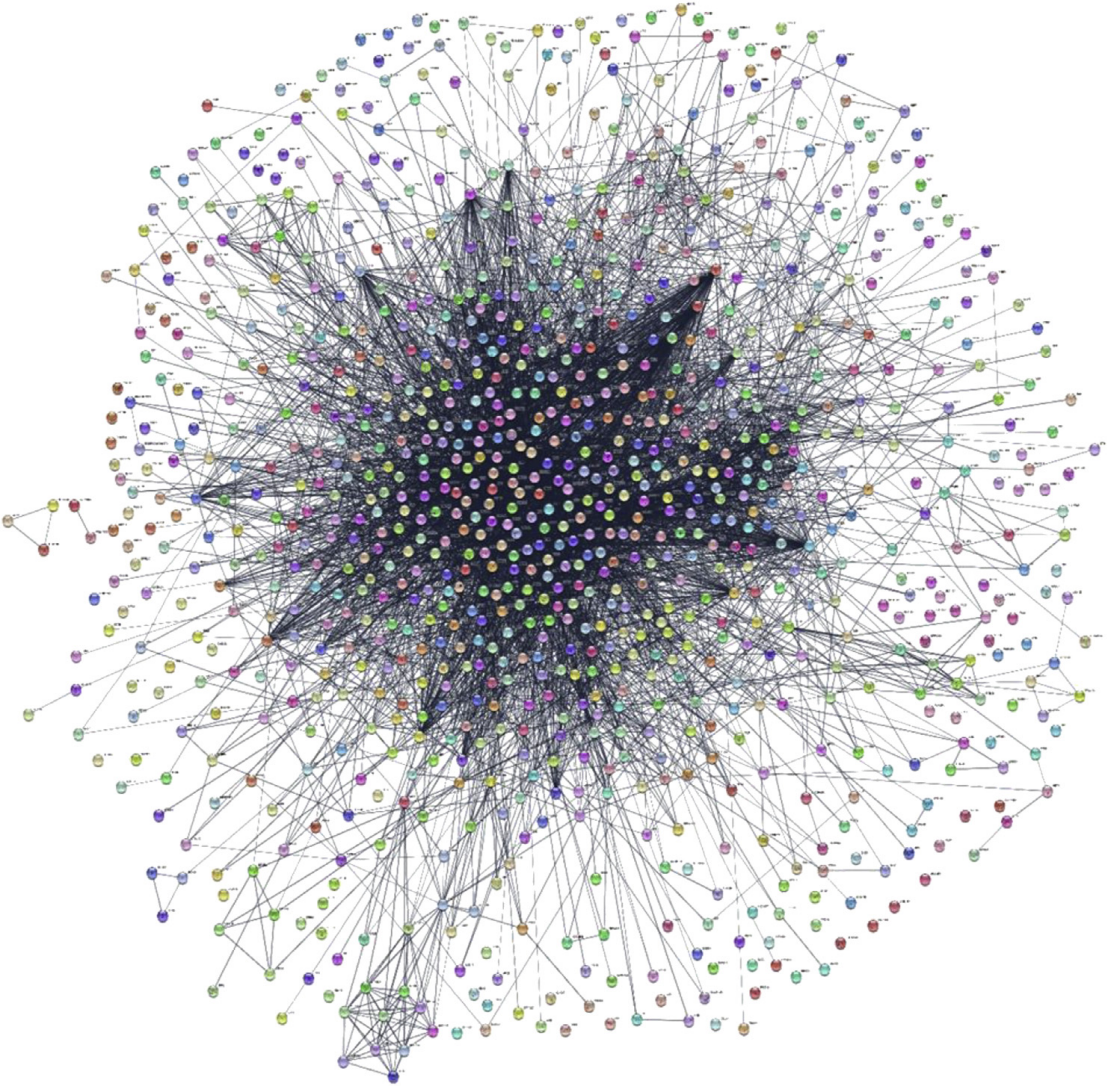
PD-associated PPI (PD-PPI) network generated with the STRING app of Cytoscape. The network includes 1000 nodes and 6851 edges.

Parkinson's disease-associated PPI (PD-PPI) network was generated using the STRING plugin of Cytoscape. The final PD-PPI network includes 1000 nodes and 6851 edges (Figure 6). Based on the network analysis through cytoHubba plugin, among five ranking methods, 44 proteins were obtained as key proteins (Figure 8a, Table 1).

**Table 1:** The top 10% genes according to 5 cytoHubba ranking methods.

The ranking methods in cytoHubba	Top 10% genes
Betweenness	FGF2, ADRB2, UBB, ADAM10, UBC, VCP, HSPA9, HSPA8, HSPA4, HSPA5, EIF2S2, ESR1, RAP1B, FOXO3, DLG4, CYC1, MBP, BDNF, DRD2, CXCL8, TAC1, NFKB1, MGEA5, CXCL12, CASP3, IL4, PTEN, IL6, ITGAM, LRRK2, GNAL, GFAP, MT-CYB, CDK5, GSK3B, ALB, LAMC2, EIF4G1, PIK3CA, YWHAZ, GAPDH, MMP9, NGF, CAT, MAPK1, MAPK8, APOA1, SNCA, MAPK3, DCTN1, CDC42, INS, RAB1A, PLG, FYN, EIF4E, RAB29, MTOR, PINK1, EGF, SOD2, SYT11, SOD1, MAPK14, APP, ACTB, TFRC, AKT1, CSNK1D, VDAC1, TP53, AR, TXN, EGR1, APOE, DECR1, HMOX1, IGF1, TH, MT-CO2, ATM, PPARGC1A, RAC1, IL10, PARP1, EDN1, MAPT, SHH, TLR4, AVP, GAK, CXCR4, PARK2, SIRT1, F2, ADCY5, PPARA, VEGFA, RPS27A, TNF
Closeness	FGF2, SH3GL2, ADRB2, UBB, ADAM10, UBC, VCP, TGFB1, HSPA8, SQSTM1, HSPA4, HSPA5, ESR1, FOXO3, FOXO1, BDNF, CXCL8, NFKB1, HSPB1, CASP8, IGF2R, CXCL12, IL4, CASP3, PTEN, IL6, PICALM, CDK5, GSK3B, ALB, BCL2L1, PIK3CA, GAPDH, YWHAZ, MMP9, NGF, CAT, MAPK1, MAPK8, TRAF6, SNCA, LRP2, MAPK3, DNAJC6, CDC42, NOS3, INS, CCL2, FYN, MTOR, PINK1, EGF, SOD2, LEP, STUB1, SOD1, MAPK14, APP, ACTB, ICAM1, TFRC, AKT1, BIN1, TP53, AR, HIF1A, EGR1, APOE, HMOX1, DECR1, IGF1, TF, TH, RAC1, IL10, EDN1, MAPT, SHH, TLR4, TLR2, AVP, GAK, CXCR4, PARK2, SIRT1, IL1B, F2, PPARG, HTT, PTGS2, GCG, CDKN2A, VEGFA, SPP1, HSPA1A, RPS27A, IGF1R, TNF, HGF, WNT5A
BottleNeck	FGF2, ADRB2, CPLX1, UBB, ADAM10, NTS, UBC, RPSA, HSPA9, HSPA8, HSPA4, FKBP4, HSPA5, EIF2S2, ESR1, DLG4, FGFR4, BDNF, DRD2, SLC40A1, CXCL8, NFKB1, DRD4, HSPB1, CASP8, ALDH2, CASP3, PTEN, IL6, ITGAM, SYNJ1, GFAP, AXIN1, MT-CYB, ALB, LAMC2, PIK3CA, YWHAZ, GAPDH, HSPD1, MMP9, THBS1, CAT, MAPK1, MAPK8, APOA1, TRAF6, SNCA, MAPK3, NCAM1, CDC42, NOS3, INS, HDAC1, RAB1A, POMC, BAX, PLG, RAB1B, EIF4E, RAB29, SOD2, SYT11, SOD1, MAPK14, APP, CCDC62, ACTB, AGFG1, TFAM, AKT1, TP53, HIF1A, TXN, OPTN, HMOX1, CYP2E1, ACE, BST1, TH, ATM, PPARGC1A, GRIN2B, RAC1, IL10, PARP1, MAPT, TLR4, TLR2, AVP, GAK, TOMM40, PARK2, ADCY5, VEGFA, NSF, RPS27A, TSC2, HGF, PARK7
Stress	FGF2, ADRB2, UBB, ADAM10, UBC, VCP, HSPA9, HSPA8, HSPA4, HSPA5, ESR1, RAP1B, FOXO3, FOXO1, DNMT1, DLG4, CYC1, MBP, BDNF, DRD2, CXCL8, NFKB1, CXCL12, CASP3, IL4, PTEN, IL6, ITGAM, LRRK2, GFAP, MT-CYB, GSK3B, ALB, EIF4G1, PIK3CA, GAPDH, HSPD1, MMP9, NGF, CAT, MAPK1, MAPK8, TRAF6, SNCA, MAPK3, DCTN1, CDC42, INS, RAB1A, POMC, PLG, FYN, RAB29, MTOR, PINK1, EGF, SOD2, SYT11, SOD1, MAPK14, APP, ACTB, AKT1, VDAC1, TP53, AR, TXN, CRHR1, EGR1, APOE, DECR1, HMOX1, IGF1, TH, MT-CO2, ATM, PPARGC1A, RAC1, IL10, PARP1, EDN1, MAPT, SHH, TLR4, AVP, SLC18A3, GAK, TOMM40, CXCR4, PARK2, SIRT1, F2, ADCY5, PPARA, GCG, VEGFA, HSPA1A, RPS27A, TNF, HGF
Radiality	FGF2, SH3GL2, ADRB2, UBB, ADAM10, UBC, VCP, TGFB1, HSPA8, SQSTM1, HSPA4, HSPA5, ESR1, FOXO3, FOXO1, BDNF, CXCL8, NFKB1, HSPB1, CASP8, IGF2R, CXCL12, IL4, CASP3, PTEN, IL6, PICALM, CDK5, GSK3B, ALB, BCL2L1, PIK3CA, GAPDH, YWHAZ, MMP9, NGF, CAT, MAPK1, MAPK8, TRAF6, SNCA, LRP2, MAPK3, DNAJC6, CDC42, NOS3, INS, CCL2, FYN, MTOR, PINK1, EGF, SOD2, LEP, STUB1, SOD1, MAPK14, APP, ACTB, ICAM1, AKT1, BIN1, TP53, AR, HIF1A, EGR1, APOE, HMOX1, DECR1, IGF2, IGF1, TF, TH, IDE, RAC1, IL10, EDN1, MAPT, SHH, TLR4, TLR2, AVP, GAK, CXCR4, PARK2, SIRT1, IL1B, PPARG, HTT, PTGS2, GCG, CDKN2A, VEGFA, SPP1, HSPA1A, RPS27A, IGF1R, TNF, HGF, WNT5A

#### 3.5.2 The key proteins extracted from the network were enriched in the programmed cell death, regulation of cell death and apoptosis

**Figure 7:**
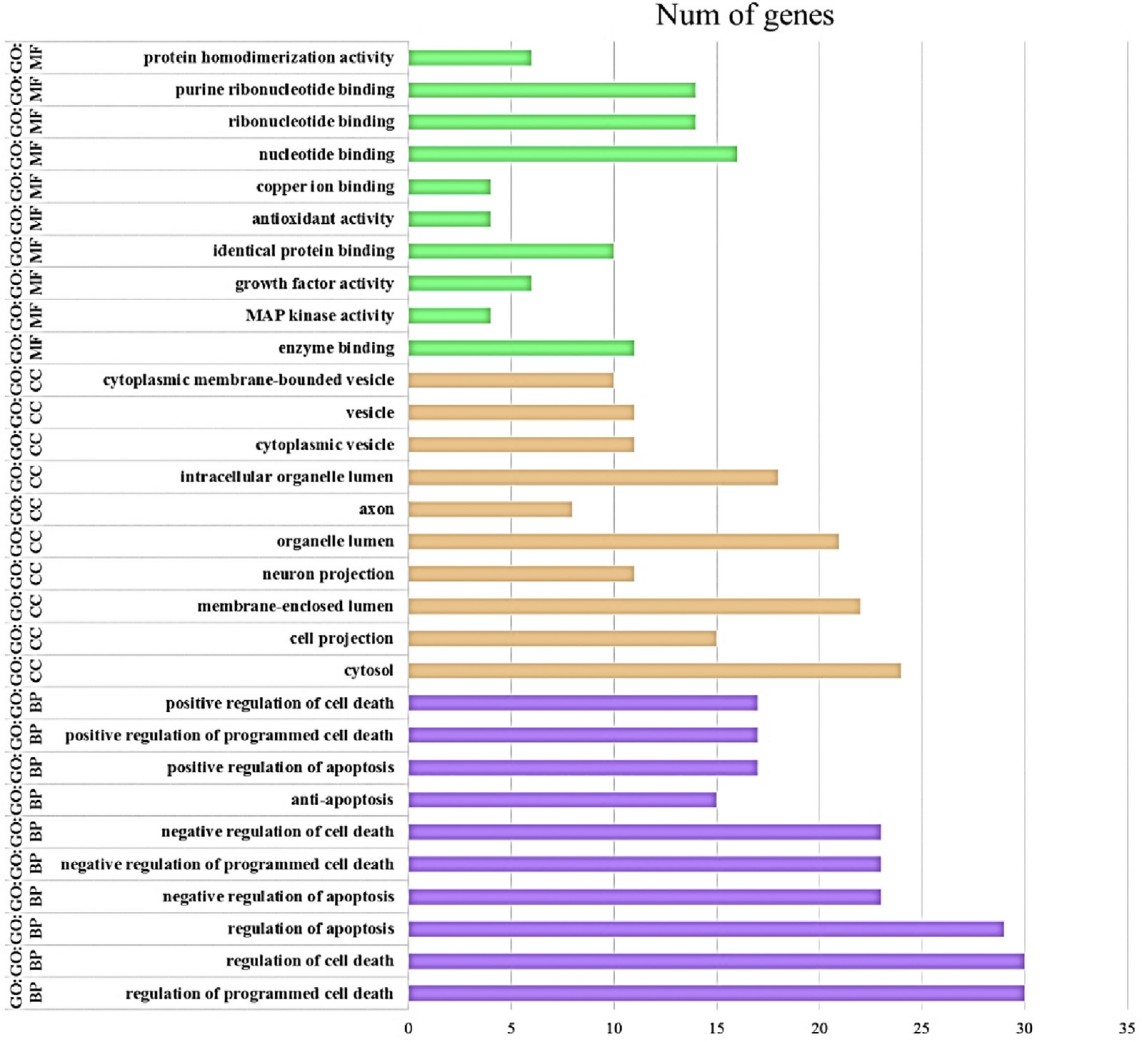
Top 10 results obtained from the GO enrichment analysis.

**Figure 8:**
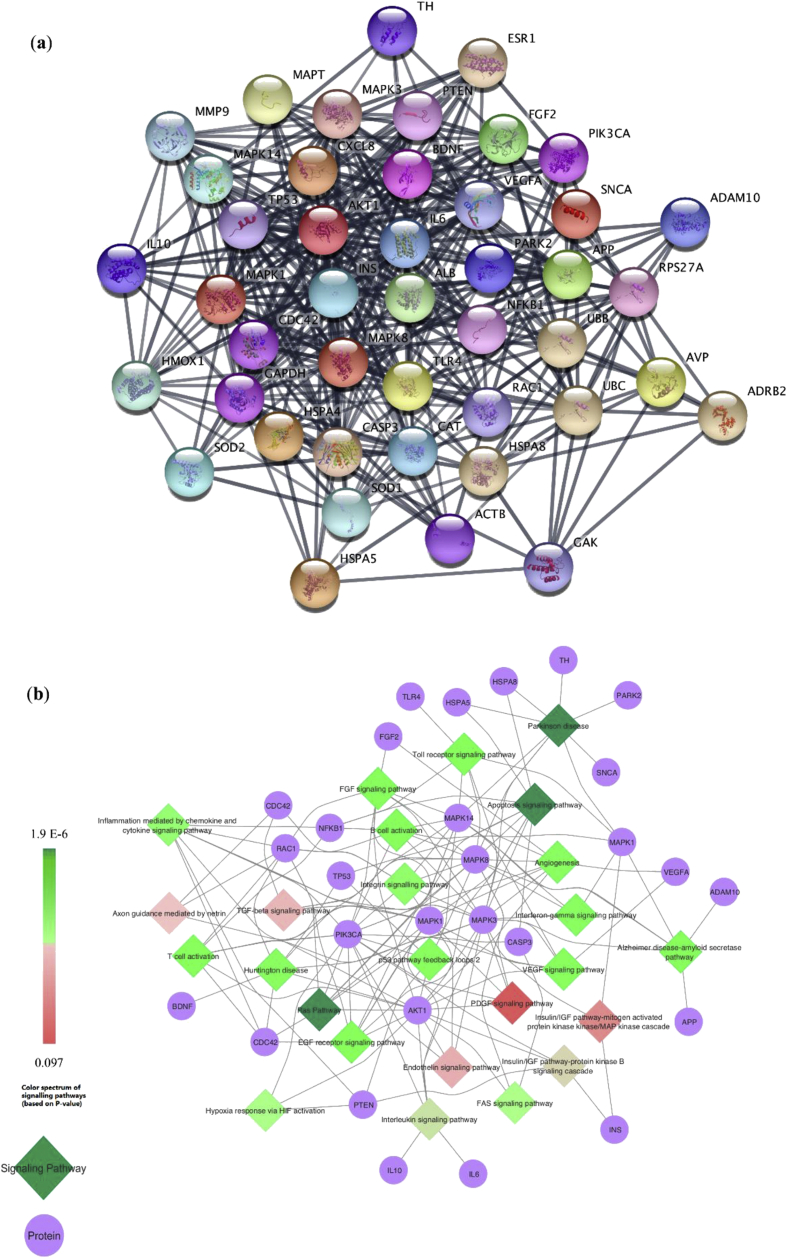
(a) The key proteins related to the PPI-PD network obtained as common proteins with the highest score through five ranking methods. (b) PANTHER pathway of the key proteins represents the signaling pathways associated with these proteins. The circle and the rhombus indicate the proteins and the signaling pathways, respectively. The color spectrum of signaling pathways varies from red (>0.05) to green (≤0.05) based on P-value.

Pathway and GO enrichment analyses of key proteins were obtained with DAVID. The top 10 results obtained from the GO enrichment analysis (according to P-value) are shown in Figure 7. The results include three parts: biological process (BP), molecular function (MF), and cell component (CC). Based on the data obtained from BP, the key proteins were significantly enriched in the regulation of programmed cell death (GO: 0043067), regulation of cell death (GO: 0010941) and regulation of apoptosis (GO: 0042981), which are all related to neuronal cell death as the major event in PD. In addition, these proteins were mainly enriched in the cytosol as CC-ontology and enzyme binding for MF-ontology. PANTHER pathways of the key proteins are shown in Figure 8b. The apoptosis signaling pathway, Parkinson's disease pathway, and Ras pathway are the most significant pathways related to these proteins. As a result, by using the network and pathway analyses, we identified key proteins in the PD-PPI network, which participate in the signaling pathways associated with PD. In the next step, to determine the neobaicalein inhibitory effect on these pathways, CASP3, TP53, MAPK14, and MAPK8 proteins were selected since the inhibition of these proteins leads to the inhibition of specified downstream signaling pathways.

#### 3.5.3 Neobaicalein could be a suitable candidate for pharmaceutical purposes

**Figure 9:**
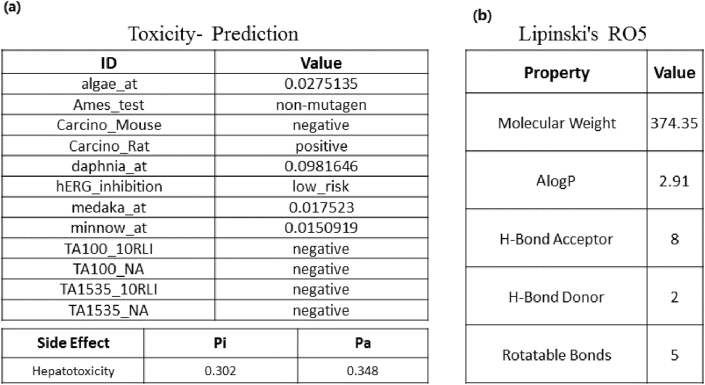
The results of (a) neobaicalein toxicity prediction and its adverse side effects using PreADMET and ADVERPred Web Services and (b) the Lipinski's RO5 of neobaicalein.

According to the data obtained from PreADME and ADVERPred, neobaicalein is a non-mutagen compound without hepatotoxicity (Figure 9a), which also obeys Lipinski's RO5 (Figure 9b).

#### 3.5.4 Docking prediction studies revealed the potential interaction of neobaicalein with MAPK14, MAPK8, and CASP3

**Figure 10:**
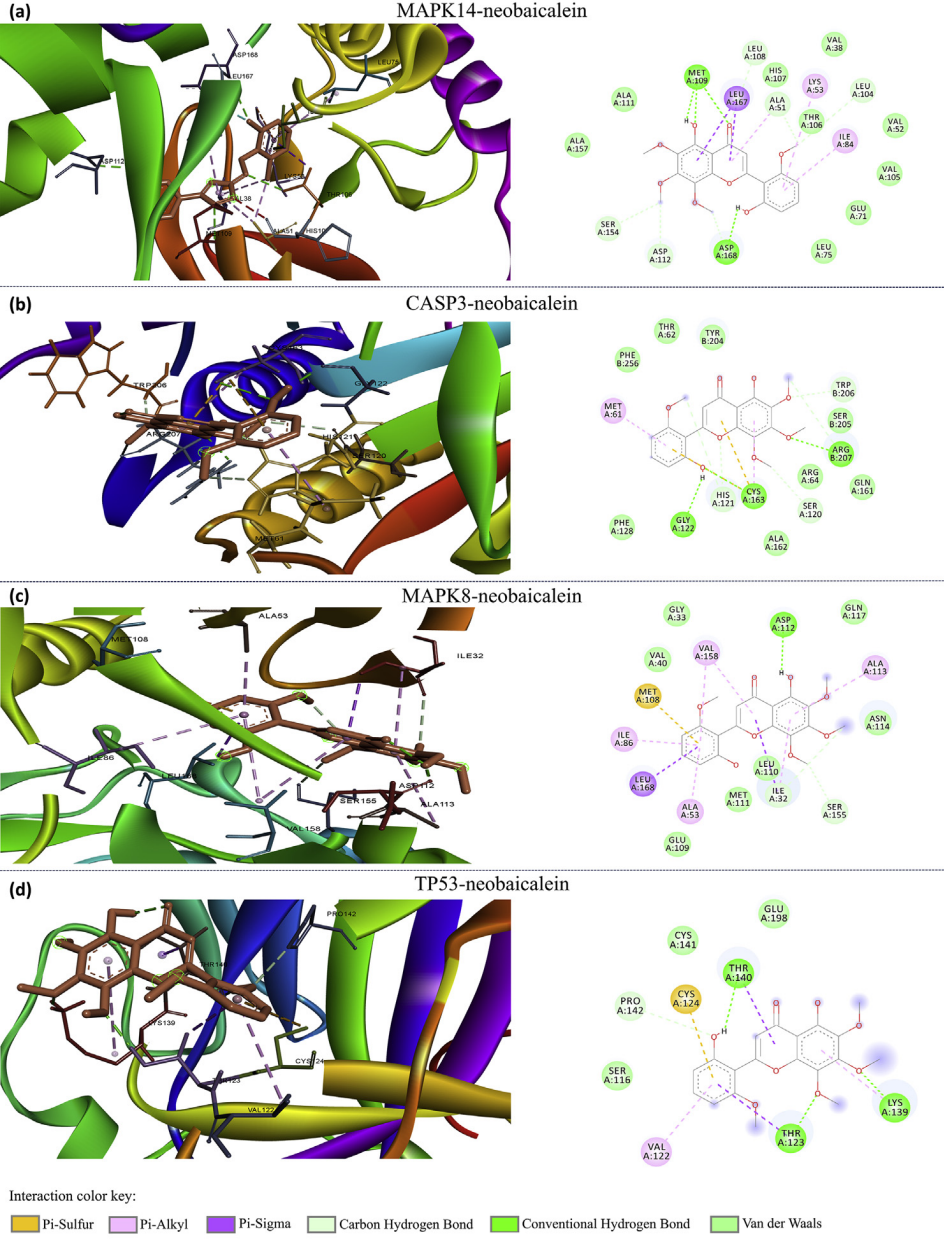
A visual illustration of the 2D (right) and 3D (left) interaction of neobaicalein with (a) MAPK14, (b) CASP3, (c) MAPK8 and (d) TP53. Amino acids involved in hydrogen bonds are shown in sharp green.

Docking simulation was performed to study the interaction of neobaicalein with the key proteins, including MAPK14 (PDB ID: 5XYY), MAPK8 (PDB ID: 2H96), CASP3 (PDB ID: 3GJQ), and TP53 (PDB ID: 1TSR) (Figure 10). Results show that neobaicalein significantly inhibits MAPK14 (lowest binding energy: -7.17 kcal/mol), CASP3 (lowest binding energy: -6.65 kcal/mol), and MAPK8 (lowest binding energy: -6.63 kcal/mol). However, neobaicalein showed less inhibitory effect against P53 (lowest binding energy: -4.51 kcal/mol). Since CASP3 and P53 are pro-apoptotic proteins [49], and the activation of MAPKs contributes to elevated oxidative stress [50], neobaicalein has the potential to inhibit the principal contributors of PD pathogenesis.

#### 3.5.5 Molecular dynamics of neobaicalein with MAPK14 and CASP3

**Figure 11:**
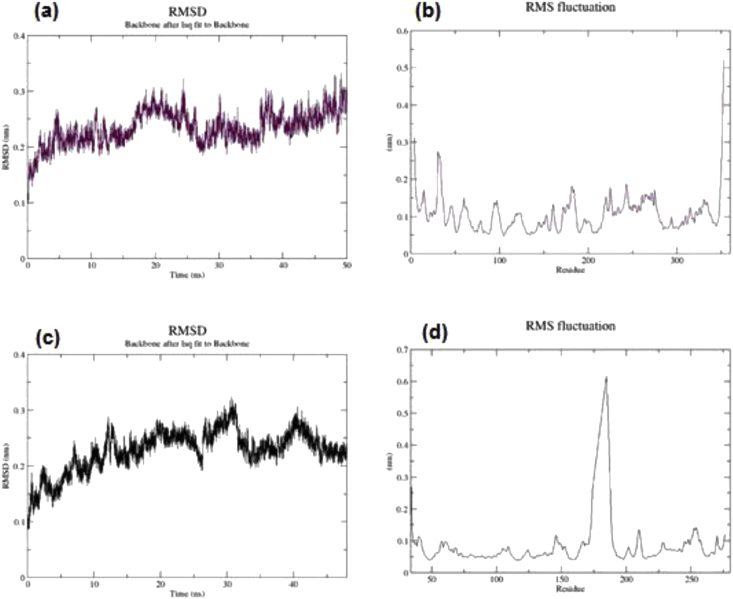
(a) RMSD and (b) RMSF plot of MAPK14-neobaicalein. (c) RMSD and (d) RMSF plot of CASP3-neobaicalein.

MD simulation was performed for 50 ns to assess neobaicalein dynamics behavior. To this end, the lowest binding energy conformation of MAPK14-neobaicalein and CASP3-neobaicalein complexes were derived, as incipient conformation for MD simulation. After the MD run, the Root Means Square Deviation (RMSD), and Root Mean Square Fluctuation (RMSF) were calculated using the GROMACS 5.0 'rmsdist' algorithm and 'rmsf' algorithm, respectively. The RMSD of MAPK14 and CASP3 with neobaicalein reached the stable configuration after 40 and 45 ns, respectively (Figure 11a, c). Moreover, RMSF of all residues, which shows the time average of RMSD for each residue, was evaluated for MAPK14 and CASP3. RMSF plot of MAPK14 in its binding form showed fluctuations of 0.15, indicating the stability of the complex (Figure 11b). The PDB structure of CASP3 has two chains, and the pick shown in the CASP3 RMSF plot is related to the region between these two chains (Figure 11d).

## 4 Discussion

Different species of *Scutellaria* are commonly used in traditional medicine due to the enrichment of high valuable secondary metabolites, especially flavonoids [30]. Since the DCMex of *S. pinnatifida* contains a considerable amount of flavonoids [34], we further analyzed this extract and purified a compound, neobaicalein, to evaluate its function. After obtaining highly purified neobaicalein, which established by TLC and NMR, its radical scavenging activity as a flavonoid compound was analyzed. There are three phenolic rings in the neobaicalein structure, which can trap free radicals as scavengers. Small molecules should be biocompatible to be used as a drug. Neobaicalein did not lyse red blood cells even at high concentrations (0.5mM), demonstrating its biocompatibility. In addition, neobaicalein is a non-mutagen compound without hepatotoxicity and obeys the RO5 (log P < 5, H-bonds donors <5, H-bonds acceptors <10, and molecular weight <500). Neobaicalein has a great opportunity to enter the market as a drug since it conforms to the RO5, and could pass clinical trials more efficiently [51]. We also explored the neuroprotective effect of neobaicalein on rotenone's toxicity, and the inflammation response in induced glial cells. Although previous studies have reported the presence of neobaicalein in *S. pinnatifida* [52, 53], no studies have yet been done on its neuroprotective effects, and according to our knowledge, this is the first report. However, there are few studies on neobaicalein remediation activity in other disorders. For instance, neobaicalein inhibits the degradation of type I collagen in human skin fibroblasts, which preserves the integrity of the extracellular matrix [54] and also prevents airway inflammation in a mouse model of asthma [55]. Additionally, neobaicalein precludes the expression of pro-protein convertase subtilisin/Kexin type 9, which avoids the recycling of low-density-lipoprotein receptors (LDLRs), and leads to the control of plasma cholesterol levels [56].

In this study, rotenone, which can cross the BBB [5] and induce neurodegeneration [57], was used to simulate an *in vitro* model of PD. In this regard, rotenone's toxicity effects on SH-SY5Y cells were analyzed in the absence and presence of different concentrations of neobaicalein. The results showed that neobaicalein considerably neutralized rotenone's neurotoxicity in 150 μM. One of the main reasons for rotenone's toxicity is its direct inhibitory effect on the mitochondrial complex I activity, which leads to dopaminergic neurodegeneration in PD [7]. Mitochondria provide ATP for the neurons and adjust the cytosolic calcium, the two necessary functions for recycling the synaptic vesicles, and the preservation of electrochemical gradients [58]. Complex I deficiency is detected in the neurons of those affected by PD, which disturbs ATP production, calcium homeostasis, and intensifies oxidative stress [8, 59]. Usually, these events lead to apoptotic neuronal death, as we also observed in the SH-SY5Y cells were treated with rotenone. However, neobaicalein pre-treatment survived the rotenone treated cells significantly and decreased the rate of early apoptosis, as well as late apoptosis/necrosis. Other flavonoids with a similar structure as neobaicalein have also been shown the potential against rotenone toxicity, suggesting the neuroprotective effects of flavonoids [9, 60, 61]. In addition to cell death, another reason, which can explain the symptoms of PD, is the disability of neurons in recruiting transmitters, synaptic communication with neighbor neurons, and glial cells. Different harmful environmental factors that disturb neuronal neurites' structure and polarization contribute to the neurodegenerative processes [62]. Experimental analysis on the hippocampal neurons revealed that rotenone administration led to inhibition of axon formation, and also reduced the length of the neurites in dopaminergic neurons [63]. Multiple lines of studies provide evidence that the inhibition of neuritogenesis by rotenone could be based on its effect on microtubule dynamics, the actin cytoskeleton, and different regulatory pathways, especially through modifying the small Rho GTPase RhoA [63]. In our experiment, rotenone diminished the number of neurites, neurite lengths, and their prevalence in differentiated SH-SY5Y cells remarkably. In the presence of neobaicalein, however, the neurites of differentiated SH-SY5Y cells remained normal remarkably.

Additionally, inflammatory events can contribute to the development of neurodegeneration by inducing the apoptotic pathways in neuronal cells [16]. Sometimes activation of glial cells leads to the initiation of the inflammatory events through the release of pro-inflammatory molecules such as NO^•^, TNF-α, IL-6, and IL-1β. In this regard, flavonoids show neuroprotective properties by inhibiting the release of pro-inflammatory cytokines through the MAPK signaling cascade [64]. The results of our study also confirmed the anti-inflammation effects of neobaicalein through the modulation of NO^•^ production induced by LPS in mixed glial cells.

Since neobaicalein neutralizes the effects of multiple factors related to PD, we decided to employ bioinformatics tools to gain knowledge on the mechanisms of its neuroprotective activities. A protein network associated with PD was identified, and its key proteins were selected. Pathway and GO enrichment analyses of the chosen proteins showed that those proteins were significantly enriched in the regulation of the programmed cell death and apoptosis-related to neuronal degeneration. In addition, based on PANTHER pathways, these proteins were associated with three pathways, including apoptosis signaling pathway, PD, and Ras pathway. Among the key proteins related to these pathways, MAPK14, CASP3, MAPK8, and TP53 were selected, as their inhibition can block downstream signaling cascades with the aim of neuroprotectivity. MAPK8 (JNK1), MAPK14 (p38α), and CASP3 have important roles in the death pathways [65, 66]. Different investigations have demonstrated that oxidative stress in dopaminergic neurons initiates the JNK and p38 pathways, which, consequently, induces apoptosis [50, 67]. Studies on the postmortem human brain have shown a correlation between the loss of dopaminergic neurons in the mesencephalon of PD patients and increased expression of CASP3 [68]. Neurotoxins, including rotenone, can activate microglial cells through the p38/MAPK pathway and induce apoptosis via JNK and CASP3 activity [69, 70, 71]. Taken together, targeting these signaling pathways may be effective in the control of PD. According to the docking analysis of this study, neobaicalein showed interactions against MAPK14, CASP3, and MAPK8. Consequently, restraining these proteins by neobaicalein gives insight into PD molecular targets for the development of therapeutic drugs. In future studies, methods such as Next Generation Sequencing can be used to assess the molecular mechanisms involved in neobaicalein protecting activity on the dopaminergic neurons.

## 5 Conclusion

We conclude that neobaicalein, a flavonoid derived from *S. pinnatifida,* could be a potential compound against the mechanisms leading to PD, such as oxidative stress, inflammation, and neurotoxins.

## Declarations

### Author contribution statement

Soha Parsafar: Performed the experiments; Analyzed and interpreted the data; Contributed reagents, materials, analysis tools or data; Wrote the paper.

Zahra Nayeri, Farhang Aliakbari, Farshad Shahi: Performed the experiments.

Mehdi Mohammadi, Dina Morshedi: Conceived and designed the experiments; Analyzed and interpreted the data.

### Funding statement

This work was supported by the National Institute of Genetic Engineering and Biotechnology (10.13039/501100006485NIGEB, Tehran, Iran) and Iran National Science Foundation (10.13039/501100003968INSF) (Grant 98003955).

### Competing interest statement

The authors declare no conflict of interest.

### Additional information

No additional information is available for this paper.

## References

[1] Caudle W.M., Guillot T.S., Lazo C.R., Miller G.W. (2012). Industrial toxicants and Parkinson's disease. Neurotoxicology.

[2] Harischandra D.S., Rokad D., Neal M.L., Ghaisas S., Manne S., Sarkar S. (2019). Manganese promotes the aggregation and prion-like cell-to-cell exosomal transmission of α-synuclein. Sci. Signal..

[3] Pan-Montojo F., Reichmann H. (2014). Considerations on the role of environmental toxins in idiopathic Parkinson's disease pathophysiology. Transl. Neurodegener..

[4] Bové J., Prou D., Perier C., Przedborski S. (2005). Toxin-induced models of Parkinson's disease. NeuroRx.

[5] Radad K., Rausch W.-D., Gille G. (2006). Rotenone induces cell death in primary dopaminergic culture by increasing ROS production and inhibiting mitochondrial respiration. Neurochem. Int..

[6] Almeida M.F., Silva C.M., D'Unhao A.M., Ferrari M.F.R. (2016). Aged Lewis rats exposed to low and moderate doses of rotenone are a good model for studying the process of protein aggregation and its effects upon central nervous system cell physiology. Arq. Neuropsiquiatr..

[7] Bhurtel S., Katila N., Srivastav S., Neupane S., Choi D.-Y. (2019). Mechanistic comparison between MPTP and rotenone neurotoxicity in mice. Neurotoxicology.

[8] Chen C., Turnbull D.M., Reeve A.K. (2019). Mitochondrial dysfunction in Parkinson's disease cause or consequence?. Biology (Basel).

[9] Ablat N., Lv D., Ren R., Xiaokaiti Y., Ma X., Zhao X. (2016). Neuroprotective effects of a standardized flavonoid extract from safflower against a rotenone-induced rat model of Parkinson's disease. Molecules.

[10] Brinkley B.R., Barham S.S., Barranco S.C., Fuller G.M. (1974). Rotenone inhibition of spindle microtubule assembly in mammalian cells. Exp. Cell Res..

[11] Meisner H.M., Sorensen L. (1966). Metaphase arrest of Chinese hamster cells with rotenone. Exp. Cell Res..

[12] Ramkumar M., Rajasankar S., Gobi V.V., Dhanalakshmi C., Manivasagam T., Justin Thenmozhi A. (2017). Neuroprotective effect of Demethoxycurcumin, a natural derivative of Curcumin on rotenone induced neurotoxicity in SH-SY 5Y Neuroblastoma cells. BMC Compl. Alternative Med..

[13] Lin D., Jing X., Chen Y., Liang Y., Lei M., Peng S. (2017). Rifampicin pre-treatment inhibits the toxicity of rotenone-induced PC12 cells by enhancing sumoylation modification of α-synuclein. Biochem. Biophys. Res. Commun..

[14] Zeng X.-S., Geng W.-S., Jia J.-J. (2018). Neurotoxin-induced animal models of Parkinson disease: pathogenic mechanism and assessment. ASN Neuro..

[15] Poewe W., Seppi K., Tanner C.M., Halliday G.M., Brundin P., Volkmann J. (2017). Parkinson disease. Nat. Rev. Dis. Prim..

[16] Gelders G., Baekelandt V., Van der Perren A. (2018). Linking neuroinflammation and neurodegeneration in Parkinson's disease. J. Immunol. Res..

[17] Kalampokini S., Becker A., Fassbender K., Lyros E., Unger M.M. (2019). Nonpharmacological modulation of chronic inflammation in Parkinson's disease: role of diet interventions. Parkinsons Dis..

[18] Bachiller S., Jiménez-Ferrer I., Paulus A., Yang Y., Swanberg M., Deierborg T. (2018). Microglia in neurological diseases: a road map to brain-disease dependent-inflammatory response. Front. Cell. Neurosci..

[19] Kim K.S., Marcogliese P.C., Yang J., Callaghan S.M., Resende V., Abdel-Messih E. (2018). Regulation of myeloid cell phagocytosis by LRRK2 via WAVE2 complex stabilization is altered in Parkinson's disease. Proc. Natl. Acad. Sci..

[20] Pohl F., Kong Thoo Lin P. (2018). The potential use of plant natural products and plant extracts with antioxidant properties for the prevention/treatment of neurodegenerative diseases: in vitro, in vivo and clinical trials. Molecules.

[21] Musthafa M.E., Bridge W., Selvaraju S., Essa M.M., Braidy N., Bridge W. (2014). Review of natural products on Parkinson's disease pathology.. J. Aging Res. Clin. Pract..

[22] Deshpande P., Gogia N., Singh A. (2019). Exploring the efficacy of natural products in alleviating Alzheimer's disease. Neural Regen. Res..

[23] Marín L., Miguélez E.M., Villar C.J., Lombó F. (2015). Bioavailability of dietary polyphenols and gut microbiota metabolism: antimicrobial properties. BioMed Res. Int..

[24] Varkuti B.H., Liu Z., Kepiro M., Pacifico R., Gai Y., Kameneka T. (2020). High-throughput small molecule screen identifies modulators of mitochondrial function in neurons. iScience.

[25] Spencer J.P.E. (2009). The impact of flavonoids on memory: physiological and molecular considerations. Chem. Soc. Rev..

[26] Mandel S., Youdim M.B. (2004). Catechin polyphenols: neurodegeneration and neuroprotection in neurodegenerative diseases. Free Radic. Biol. Med..

[27] Elbaz A., Carcaillon L., Kab S., Moisan F. (2016). Epidemiology of Parkinson's disease. Rev. Neurol. (Paris).

[28] de Andrade Teles R.B., Diniz T.C., Costa Pinto T.C., de Oliveira Júnior R.G., Gama e Silva M., de Lavor É.M. (2018). Flavonoids as therapeutic agents in alzheimer's and Parkinson's diseases: a systematic review of preclinical evidences. Oxid. Med. Cell Longev..

[29] KH (1982). R. Flora Iranica. Graz.

[30] Shang X., He X., He X., Li M., Zhang R., Fan P. (2010). The genus Scutellaria an ethnopharmacological and phytochemical review. J. Ethnopharmacol..

[31] A M V (1996). Dictionary of Iranian Plant Names.

[32] Nishikawa K., Furukawa H., Fujioka T., Fujii H., Mihashi K., Shimomura K. (2000). Phenolics in tissue cultures of Scutellaria. Recent Res. Dev. Phytochem..

[33] Awad R., Arnason J.T., Trudeau V., Bergeron C., Budzinski J.W., Foster B.C. (2003). Phytochemical and biological analysis of Skullcap (Scutellaria lateriflora L.): a medicinal plant with anxiolytic properties. Phytomedicine.

[34] Sashourpour M., Zahri S., Radjabian T., Ruf V., Pan-Montojo F., Morshedi D. (2017). A study on the modulation of alpha-synuclein fibrillation by Scutellaria pinnatifida extracts and its neuroprotective properties. PloS One.

[35] Jaiteh M., Zeifman A., Saarinen M., Svenningsson P., Bréa J., Loza M.I. (2018). Docking screens for dual inhibitors of disparate drug targets for Parkinson's disease. J. Med. Chem..

[36] Wang W., Xiong X., Li X., Zhang Q., Yang W., Du L. (2019). In silico investigation of the anti-tumor mechanisms of epigallocatechin-3-gallate. Molecules.

[37] Aliakbari F., Shabani A.A., Bardania H., Mohammad-Beigi H., Tayaranian Marvian A., Dehghani Esmatabad F. (2018). Formulation and anti-neurotoxic activity of baicalein-incorporating neutral nanoliposome. Colloids Surf. B Biointerfaces.

[38] Aliakbari F., Mohammad-Beigi H., Rezaei-Ghaleh N., Becker S., Dehghani Esmatabad F., Eslampanah Seyedi H.A. (2018). The potential of zwitterionic nanoliposomes against neurotoxic alpha-synuclein aggregates in Parkinson's Disease. Nanoscale.

[39] Degl'Innocenti D., Ramazzotti M., Sarchielli E., Monti D., Chevanne M., Vannelli G.B. (2019). Oxadiazon affects the expression and activity of aldehyde dehydrogenase and acylphosphatase in human striatal precursor cells: a possible role in neurotoxicity. Toxicology.

[40] Påhlman S., Ruusala A.-I., Abrahamsson L., Mattsson M.E.K., Esscher T. (1984). Retinoic acid-induced differentiation of cultured human neuroblastoma cells: a comparison with phorbolester-induced differentiation. Cell Differ..

[41] Morshedi D., Nasouti M. (2016). Essential oils may lead α -synuclein towards toxic fibrils formation. Parkinsons Dis..

[42] Meijering E., Jacob M., Sarria J.-C.F., Steiner P., Hirling H., Unser M. (2004). Design and validation of a tool for neurite tracing and analysis in fluorescence microscopy images. Cytometry.

[43] Heravi M., Dargahi L., Parsafar S., Tayaranian Marvian A., Aliakbari F., Morshedi D. (2019). The primary neuronal cells are more resistant than PC12 cells to α-synuclein toxic aggregates. Neurosci. Lett..

[44] Griess P. (1879). Bemerkungen zu der Abhandlung der HH. Weselsky und Benedikt “Ueber einige Azoverbindungen”. Berichte der Dtsch Chem Gesellschaft.

[45] Lipinski C.A., Lombardo F., Dominy B.W., Feeney P.J. (1997). Experimental and computational approaches to estimate solubility and permeability in drug discovery and development settings. Adv. Drug Deliv. Rev..

[46] Ivanov S.M., Lagunin A.A., Rudik A.V., Filimonov D.A., Poroikov V.V. (2018). ADVERPred–web service for prediction of adverse effects of drugs. J. Chem. Inf. Model..

[47] Mohammadi A., Asili J., Emami S., Mighani H., Bibak B. (2013). Phytochemical investigation on Scutellaria pinnatifida roots. J. N. Khorasan Univ. Med. Sci..

[48] Guo W., Stoklund Dittlau K., Van Den Bosch L. (2020). Axonal transport defects and neurodegeneration: molecular mechanisms and therapeutic implications. Semin. Cell Dev. Biol..

[49] Zhao R., Kaakati R., Lee A.K., Liu X., Li F., Li C.-Y. (2018). Novel roles of apoptotic caspases in tumor repopulation, epigenetic reprogramming, carcinogenesis, and beyond. Canc. Metastasis Rev..

[50] Jha S.K., Jha N.K., Kar R., Ambasta R.K., Kumar P. (2015). p38 MAPK and PI3K/AKT signalling cascades inParkinson's disease. Int. J. Mol. Cell Med..

[51] Lipinski C.A. (2004). Lead- and drug-like compounds: the rule-of-five revolution. Drug Discov. Today Technol..

[52] Tayarani-Najarani Z., Asili J., Parsaee H., Mousavi S.H., Mashhadian N.V., Mirzaee A. (2012). Wogonin and neobaicalein from Scutellaria litwinowii roots are apoptotic for HeLa cells. Rev. Bras. Farmacogn.

[53] Boozari M., Mohammadi A., Asili J., Emami S.A., Tayarani-Najaran Z. (2015). Growth inhibition and apoptosis induction by Scutellaria pinnatifida A. Ham. on HL-60 and K562 leukemic cell lines. Environ. Toxicol. Pharmacol..

[54] Lee Y.H., Seo E.K., Lee S.-T. (2019). Skullcapflavone II inhibits degradation of type I collagen by suppressing MMP-1 transcription in human skin fibroblasts. Int. J. Mol. Sci..

[55] Jang H.-Y., Ahn K.-S., Park M.-J., Kwon O.-K., Lee H.-K., Oh S.-R. (2012). Skullcapflavone II inhibits ovalbumin-induced airway inflammation in a mouse model of asthma. Int. Immunopharm..

[56] Nhoek P., Chae H.-S., Masagalli J., Mailar K., Pel P., Kim Y.-M. (2018). Discovery of flavonoids from Scutellaria baicalensis with inhibitory activity against PCSK 9 expression: isolation, synthesis and their biological evaluation. Molecules.

[57] Lin T.-K., Chen S.-D., Chuang Y.-C., Lin H.-Y., Huang C.-R., Chuang J.-H. (2014). Resveratrol partially prevents rotenone-induced neurotoxicity in dopaminergic SH-SY5Y cells through induction of heme oxygenase-1 dependent autophagy. Int. J. Mol. Sci..

[58] Devine M.J., Kittler J.T. (2018). Mitochondria at the neuronal presynapse in health and disease. Nat. Rev. Neurosci..

[59] Park J.-S., Davis R.L., Sue C.M. (2018). Mitochondrial dysfunction in Parkinson's disease: new mechanistic insights and therapeutic perspectives. Curr. Neurol. Neurosci. Rep..

[60] Elmazoglu Z., Yar Saglam A.S., Sonmez C., Karasu C. (2020). Luteolin protects microglia against rotenone-induced toxicity in a hormetic manner through targeting oxidative stress response, genes associated with Parkinson's disease and inflammatory pathways. Drug Chem. Toxicol..

[61] Maher P. (2017). Protective effects of fisetin and other berry flavonoids in Parkinson's disease. Food Funct..

[62] Bisbal M., Sanchez M. (2019). Neurotoxicity of the pesticide rotenone on neuronal polarization: a mechanistic approach. Neural Regen. Res..

[63] Sanchez M., Gastaldi L., Remedi M., Cáceres A., Landa C. (2008). Rotenone-induced toxicity is mediated by rho-GTPases in hippocampal neurons. Toxicol. Sci..

[64] Ginwala R., Bhavsar R., Chigbu D.I., Jain P., Khan Z.K. (2019). Potential role of flavonoids in treating chronic inflammatory diseases with a special focus on the anti-inflammatory activity of apigenin. Antioxidants.

[65] Bohush A., Niewiadomska G., Filipek A. (2018). Role of mitogen activated protein kinase signaling in Parkinson's disease. Int. J. Mol. Sci..

[66] Alnemri E.S., Livingston D.J., Nicholson D.W., Salvesen G., Thornberry N.A., Wong W.W. (1996). Human ICE/CED-3 protease nomenclature. Cell.

[67] Dzamko N., Zhou J., Huang Y., Halliday G.M. (2014). Parkinson's Disease-implicated kinases in the brain; insights into disease pathogenesis. Front. Mol. Neurosci..

[68] Hartmann A., Hunot S., Michel P.P., Muriel M.-P., Vyas S., Faucheux B.A. (2000). Caspase-3: a vulnerability factor and final effector in apoptotic death of dopaminergic neurons in Parkinson's disease. Proc. Natl. Acad. Sci..

[69] Chun H.S., Gibson G.E., DeGiorgio L.A., Zhang H., Kidd V.J., Son J.H. (2001). Dopaminergic cell death induced by MPP+, oxidant and specific neurotoxicants shares the common molecular mechanism. J. Neurochem..

[70] Peng J., Andersen J. (2003). The role of c-jun N-terminal kinase (JNK) in Parkinson's disease. IUBMB Life.

[71] Dodel R., Du Y., Bales K., Ling Z.-D., Carvey P., Paul S. (1998). Peptide inhibitors of caspase-3-like proteases attenuate MPP+ -induced toxicity of cultured fetal rat mesencephalic dopamine neurons. Neuroscience.

