# Supplementary material for: Multiple neuroprotective features of Scutellaria pinnatifida–derived small molecule
Source: Heliyon. 2020 Aug 28;6(8):e04737. doi: 10.1016/j.heliyon.2020.e04737 (PMC7472859; doi:10.1016/j.heliyon.2020.e04737)
Supplement: Supplementary material [file mmc1.docx]

**Supplementary material**

**Multiple neuroprotective features of *Scutellaria Pinnatifida*–derived small molecule**

Soha Parsafar ^1^, Zahra Nayeri ^1^, Farhang Aliakbari ^1^, Farshad Shahi ^1^, Mehdi Mohammadi **^*^**^1^, Dina Morshedi ^*1^

1. Department of Bioprocess Engineering, Institute of Industrial and Environmental Biotechnology, National Institute of Genetic Engineering and Biotechnology, Tehran, Iran.

*** Corresponding Authors:**

[morshedi@nigeb.ac.ir](mailto:morshedi@nigeb.ac.ir), National Institute of Genetic Engineering and Biotechnology (NIGEB), Address: Shahrak-e Pajoohesh, km 15 Tehran - Karaj Highway, Tehran, Iran, P.O.Box:14965/161, Tehran, Iran.

[M.mohammadi@nigeb.ac.ir](mailto:M.mohammadi@nigeb.ac.ir), National Institute of Genetic Engineering and Biotechnology (NIGEB), Address: Shahrak-e Pajoohesh, km 15 Tehran - Karaj Highway, Tehran, Iran, P.O. Box: 14965/161, Tehran, Iran


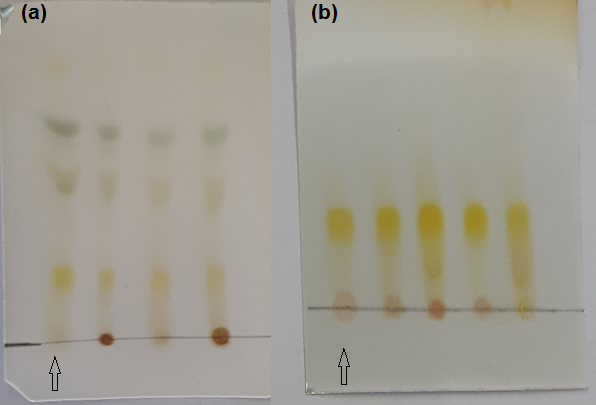


**Figure S1.** Isolation pattern of DCMex (a) and neobaicalein (b) on TLC. The samples shown in the first lines (arrows) are the fractions selected for the next steps of the experiments.
